# Perceptions of incarcerated people: prison conditions, public health, and justice in the United States

**DOI:** 10.1057/s41271-024-00496-z

**Published:** 2024-07-02

**Authors:** Jordan M. Hyatt, Synøve N. Andersen, Britte van Tiem

**Affiliations:** 1 Department of Criminology and Justice Studies and Center for Public Policy, Drexel University, Philadelphia, PA USA; 2 Department of Sociology and Human Geography, University of Oslo, Oslo, Norway; 3 Department of Criminology & Criminal Justice and School of Public Policy, University of Maryland, College Park, MD USA

**Keywords:** Prison climate, Survey research, Prison health, Incarceration

## Abstract

Carceral conditions in the United States may serve as a proxy for crises within justice and health systems. This study seeks to consider and measure prison climate from the perspective of incarcerated people. By examining within-facility differences in carceral experiences, results shed light on the complex nexus between the carceral context, health, and justice. We administered the Prison Climate Questionnaire (PCQ) to the complete population of incarcerated men in a correctional facility located in the Eastern United States. In this facility, housing units hold distinct populations, fulfill different functions, and can offer unique programming. We regress select items from the PCQ on a set of dummies corresponding to different residential units within the facility. Responses indicate low but relatively uniform perceptions of overall personal health, as well as access to, and satisfaction with, medical care. Between-unit differences emerge regarding staff relationships, experiences of discrimination, and levels of isolation. The perspectives of incarcerated people can, and should, play a role in understanding and conceptualizing the nature of the prison environment. Policy responses, especially those that impact the health and well-being of currently and formerly incarcerated people, can be informed by these perspectives.

## Key messages

- Administrative data on prison climate and conditions omit or fail to measure many factors relevant to understanding the health and well-being of people in prison.
- Surveys of incarcerated people provide important data and context, give voice to a group that is often marginalized in health and justice research, and provide actionable insight into carceral policy.

## Introduction

There are more people in prison in the United States (US) than in almost any other country in the world [1]. The effects of incarceration on people and communities are profound, especially for those already marginalized by high rates of poverty and lack of access to services and opportunities [2]. The impact on families [3, 4], economic well-being [5], and the social fabric of communities [6] can create a feedback loop that perpetuates a cycle of victimization, crime, and imprisonment [7]. While much of the complex social and justice systems that facilitate the cycling of people in and out of prisons have been critically examined, the nature and impacts of the carceral environment remain less understood. Similarly, research shows that the well-being of incarcerated people is often meaningfully worse than that of a comparable, non-imprisoned population [8, 9]. Classical theoretical works connect the experience of incarceration with physical and mental health [10, 11], yet key elements of that relationship remain unobserved. This leaves a foundational gap in the information needed to develop evidence-based correctional and health policy.

One of the primary purposes of incarceration is to reduce crime and improve public safety through deterrence, incapacitation, and rehabilitation. Whether a correctional system is successful in achieving this goal has long been a primary area of inquiry for criminologists worldwide, and the bulk of research across a range of carceral contexts suggests that imprisonment alone has a very limited impact on post-release offending [12]. More recent scholarship has, however, highlighted many *unintended* consequences of imprisonment on individuals and communities [13], including the strong impact of imprisonment on the health of currently and formerly incarcerated people. Social isolation, exposure to infection, and myriad forms of social and physical stress experienced in prison can be associated with a variety of longer-term, negative health outcomes including cardiovascular issues, infectious diseases, and mental health problems [14–16]. Many factors impacting health in the community also cross prison walls or have influential parallels or proxies within those facilities. For example, ensuring access to medical care for marginalized or at-risk communities is a challenge both in the community and within prisons and jails [17, 18]. Also, racism and discrimination, increasingly recognized as issues of public health, can reflect the actions of correctional staff and of other incarcerated people.

Although researchers increasingly recognize the importance of specific interventions or aspects of incarceration for the health of individuals and, in turn, communities [15, 19, 20], the potential impact of the broader prison environment and the lived experience of people in prison in shaping such a relationship remains poorly understood [21]. What happens in prison, especially on a day-to-day basis, remains more or less an empirical “black box,” thus creating a bi-directional gap in understanding. Access to data about prisons and incarcerated people is often restricted; concerns about security and privacy prevail and administrative records available to researchers or policymakers are often limited. The result is an imperfect and potentially skewed perspective on the health of incarcerated people and the nature prison environment [22].

To better understand the impact of incarceration on public health, it is essential to broaden the scope of data collected about incarcerated people to include information on health as well as their subjective experiences in the prison environment. We explore the relevance of the concept of prison climate, a multidimensional construct used in criminology and prison sociology that incorporates elements of the built environment and the social, relational, and organizational dynamics that play out within prison walls, as a viable approach to facilitating efforts to empirically examine the lived experience of incarceration [23]. Research has linked prison climate to behavior [23], recidivism [24], mental health [25], and access to medical care at the individual level [26]. Others have highlighted that survey measures are particularly useful at capturing shared experiences that become meaningful in analyses at the prison or unit level [27]. This has opened up new, comparative ways to frame inquiries into the diverse health profiles of carceral populations and the impact of the prison environment on health itself [28, 29]. Notably, climate surveys have utility for understanding the potentially problematic and encouraging elements of the carceral atmosphere at the micro- and macro-level, shedding light on areas of the prison environment that have long gone unobserved.

## Data and methods

### The Prison Climate Questionnaire (PCQ)

Several instruments exist in criminology, penology, and related fields to quantify the aspects of the social and physical prison environment [30]. The Prison Climate Questionnaire (PCQ) is a relatively new climate assessment instrument developed as part of a long-term, nationwide effort to examine prison life in The Netherlands [31]. The PCQ focuses on six key domains relevant to the experience of incarceration: autonomy, safety and order, relationships in prison, meaningful activities, contact with the outside world, and the quality of facilities.

Although the PCQ was originally designed to assess the overall quality of life in prison, the instrument covers dimensions that have a clear and direct link to public health. It taps into the subjective experiences of physical and mental health of people *while they are in prison* and explores elements of the prison environment that can impact both their short- and long-term health outcomes—including access to fresh air and nutritious food, perceived levels of stress and safety, and meaningful human interaction [32]. This study draws on select items from the PCQ with relevance to public health and thus does not use the scales included in the original survey. Psychometric analyses of the full instrument using data from the Netherlands, as well as the data used for this study, support the use of the PCQ across contexts [33, 34]. 

Self-reported data, as generated through the PCQ, on health gathered in prison climate surveys can fill gaps in administrative data and illuminate corners of the prison experience that are often overlooked, unmeasured, or disregarded in studies of mass incarceration and public health.

### An administration of the PCQ in the US

To measure prison climate in the United States, we adapted the Dutch version of the PCQ before data collection began. Minor adjustments were made to the questionnaire to ensure its relevance to the US context. For example, changes were made to language and job titles in the prison to fit the American setting. Project-focused items were also added to supplement the survey.

Our research team administered the survey in 2022 at a facility located in a large, Northeastern state. It has a capacity for approximately 1,200 men across 14 housing units that differ in terms of staffing, target population, and institutional design. On the General Population (GP) units, incarcerated people have no specialized programming or resources. Two men inhabit each cell, typically in units of 128 men. In the Therapeutic Community (TC) units, housing conditions are similar to the GP units, with the exception that the men participate in a mandatory, full-time drug treatment program. Residence on the Honor Block (HB) must be earned through a history of productive engagement within the prison community and a recent history free of misconduct. Housing on this block offers additional privileges, including extra time out of cells, access to fitness and recreational equipment, and a dog and cat fostering program. We consider one more unit, the Restrictive Housing Unit (RHU), that prison authorities use as a sanction or during an active investigation for serious rule violations. On this unit, incarcerated people are only permitted out for the mandatory amount of out-of-cell exercise time. The prison largely restricts visits for individuals in the RHU (except legal counsel), and they can access few services or programs offered in the facility. To some extent, unit designations overlap with custody levels. Individuals on the Honor Block need to be at custody level 3 or lower. The RHU disproportionately, but not exclusively, houses individuals at higher custody levels. The GP and TC units house individuals from all custody levels by design. This analysis focuses on prison housing units rather than the overall facility as incarcerated people spend a significant amount of time there, and relevant health and climate indicators may vary most meaningfully at this level due to fundamental, and intentional, unit-level distinctions in conditions of confinement.

Assignment to types of housing is not random. Prison officials assign individuals to a class of housing unit based on the factors related to security requirements, treatment needs, and/or as an incentive/reward for positive behaviors. Although an incarcerated person can be, and often is assigned to several facilities and units throughout a sentence, the PCQ items ask for responses about the unit on which the individual was living at the time of administration of the survey.

### Participants

Over the course of two weeks, the research team provided every individual housed at the facility the opportunity to complete the PCQ. Research team members went to every housing unit at least once; we did not conduct any individual-level or targeted recruitment activities. During the consent and administration processes, research team members explained the nature of the survey, and handed each incarcerated man a copy of the pencil-and-paper instrument in their cell. It included instructions, the instrument, and a copy of the informed consent form, all of which could be completed while in their cell. Independent research team members remained on or near the unit and collected completed and blank surveys directly from the respondents. If any individual requested additional time to complete the survey, a research team member asked him to keep the document securely in the cell and informed him that a member of the research team would return the following day to collect the survey.

Participation in the survey was fully voluntary and the research team provided nominal compensation to each respondent for their time, so long as the individual returned a survey, irrespective of its completeness. At the time of administration, 973 individuals were incarcerated in the facility, and, after the survey period, we had received 623 unique responses. This translates to an overall response rate of 64 percent. 

Unit-specific response rates ranged from 56 percent (HB) to 73 percent (RHU). The respondents in the all-male sample averaged just over 38 years of age, and 38 percent identified as Black [34]. We manually coded de-identified survey records into a secure database for analysis.

### Statistical analysis

In this facility, as is common, housing units may hold different populations, fulfill different functions, and offer unique programming. We might, therefore, expect units to have distinct climates, including different health-relevant characteristics. This provides an opportunity to explore both the perspectives of incarcerated people and the relevance of the PCQ, particularly through a public health lens. In the analysis that follows, we regress select items from the PCQ on a set of dummies corresponding to the units discussed above using Ordinary Least Squares regression. General Population units serve as the reference unit, the natural baseline for comparisons of climate as those units reflect standard, unspecialized conditions of confinement.

To explore between-unit variation in relevant outcomes we regress questionnaire items on unit type in a simple linear regression model. We base regressions on data from 564 respondents, some housed on the General Population units (*N* = 348), others on Therapeutic Community units (*N* = 133), the Honor Block (*N* = 63), or the Restricted Housing Unit (*N* = 20) during the time of survey administration. We did not include individuals held in the Infirmary on the Recovery Unit or on the Transitional Housing Unit in this analysis due to the more temporary and transient nature of the populations of these units.

## Results

Table 1, panel A, includes select items from the PCQ that provide insight into health-related factors in the carceral environment. The responses indicate low but relatively uniform perceptions of overall personal health, access to, and satisfaction with medical care. This pattern likely mirrors the relatively poor health in this population and a situation where prisons provided health services uniformly at the facility level. Differences emerge regarding access to exercise facilities, with a minority of RHU respondents reporting having used them over the past month as incarcerated people cannot access facility-standard recreational facilities while assigned to this unit. RHU respondents also reported more disruptions to their sleep; people on the HB, the least restricted unit, reported the best quality of sleep.

**Table 1:** Results from OLS regression analyses of health outcomes and prison outcomes on unit type

Questionnaire Item	(Intercept)	TC	Hons	RHU
***Panel A: Health Outcomes***				
**Access to and satisfaction with medical care**				
1. I can get medical care here if I want to	3.087***	0.128	-0.184	-0.187
	(0.058)	(0.111)	(0.149)	(0.249)
2. Health problems are being taken care of adequately here	2.588***	0.260*	-0.120	-0.388
	(0.062)	(0.118)	(0.159)	(0.265)
3. I am satisfied with the work of the doctor	2.540***	0.311*	0.010	-0.440 +
	(0.064)	(0.121)	(0.162)	(0.266)
**Physical health, sleep, and exercise**				
4. My physical health is good	3.446***	0.165	0.135	-0.146
	(0.058)	(0.110)	(0.148)	(0.246)
5. In the past month, did you use the sports facilities?	0.745***	0.049	-0.003	-0.345***
	(0.023)	(0.044)	(0.060)	(0.099)
6. Due to poor conditions in this institution and/or my cell, I can't sleep well	3.018***	-0.018	-0.318*	0.482 +
	(0.062)	(0.117)	(0.159)	(0.262)
**Mental health**				
7. During the past week, did you feel… nervous?	0.559***	0.115*	-0.027	0.241*
	(0.026)	(0.050)	(0.067)	(0.112)
8. During the past week, did you feel… hopeless?	0.472***	-0.068	-0.117 +	0.228*
	(0.027)	(0.051)	(0.068)	(0.114)
9. During the past month, did you feel… happy?	0.874***	0.034	0.093*	-0.124 +
	(0.017)	(0.032)	(0.043)	(0.072)
**Victimization**				
10. In the past month, have you been yelled at or threatened by a fellow incarcerated person?	0.228***	0.010	-0.051	0.172 +
	(0.023)	(0.043)	(0.058)	(0.097)
11. In the past month, have you been yelled at or threatened by a staff member?	0.510***	0.021	-0.220**	0.240*
	(0.027)	(0.051)	(0.068)	(0.114)
12. In the past month, have you been punched, pushed, or kicked by an incarcerated person?	0.091***	-0.006	0.006	0.109
	(0.016)	(0.030)	(0.040)	(0.067)
13. In the past month, have you been punched, pushed, or kicked by a staff member?	0.029**	0.024	0.050 +	0.121*
	(0.011)	(0.021)	(0.028)	(0.047)
***Panel B: Prison Outcomes***				
**Personal relationships and support in prison**				
14. Incarcerated people on this unit treat each other respectfully	3.078***	0.112	0.525***	-0.478*
	(0.052)	(0.100)	(0.133)	(0.224)
15. Incarcerated people on this unit help and support each other	2.776***	0.459***	0.637***	-0.226
	(0.052)	(0.098)	(0.132)	(0.221)
16. Staff on this unit help me if I have problems	2.610***	0.094	1.024***	-0.360
	(0.059)	(0.113)	(0.151)	(0.254)
17. Staff on this unit motivate and encourage me to participate in activities	2.116***	0.376***	1.011***	-0.266
	(0.059)	(0.112)	(0.150)	(0.252)
18. Staff on this unit treat me with respect	2.612***	0.153	1.261***	-0.262
	(0.063)	(0.120)	(0.161)	(0.270)
**Support system and contact with the outside**				
19. I have a good support system on the outside	3.991***	0.244*	0.251	-0.141
	(0.061)	(0.116)	(0.157)	(0.261)
20. In the past month, have you called your family, friends, or partner?	0.942***	0.005	0.010	-0.492***
	(0.013)	(0.025)	(0.034)	(0.056)
21. In the past month, did you receive a visit?	0.451***	-0.023	0.194**	-0.151
	(0.027)	(0.051)	(0.068)	(0.114)
**Safety and discrimination**				
22. I feel safe in this institution	3.623***	-0.004	0.361**	-0.273
	(0.052)	(0.098)	(0.133)	(0.220)
23. I feel safe on my unit	3.748***	0.063	0.285*	-0.398*
	(0.047)	(0.089)	(0.120)	(0.199)
24. I feel safe in my cell	4.000***	0.137	0.098	-0.350 +
	(0.044)	(0.084)	(0.114)	(0.189)
25. I am afraid of some incarcerated people	2.026***	0.042	0.121	0.274
	(0.059)	(0.111)	(0.150)	(0.249)
26. I am afraid of some staff	2.513***	-0.036	-0.087	0.737*
	(0.069)	(0.131)	(0.177)	(0.293)
27. In the past month, I have been discriminated by a staff member on this unit	0.487***	0.044	-0.390***	0.069
	(0.026)	(0.050)	(0.067)	(0.117)
28. In the past month, I have been discriminated by an incarcerated person on this unit	0.296***	0.032	-0.151*	-0.138
	(0.024)	(0.046)	(0.062)	(0.106)

Regression based on data from 564 respondents who were housed on the General Population unit (*N* = 348), Therapeutic Community unit (*N* = 133), the Honor Block (*N* = 63), or the Restricted Housing Unit (*N* = 20) during the time of survey administration. Individuals in General population are the reference category. Individuals held in the Infirmary on the Recovery Unit and Transitional Housing Unit are not included in this analysis due to the more transient nature of the populations on these units. Items 1–4, 6,14–19, and 22–26 are five-point Likert Scale items. The other items use a variety of scales on the original survey, which have been converted into binary responses for this analysis. F-tests on the joint significance of the coefficients are not significant for questions 1, 4, 10, 12, 19, 25, and 26. They are significant for questions 2, 3, 6–9, 13, 21–24, and 28 (at the .05 level of significance) and for questions 5, 11, 14–18, 20, and 27 (at the .01 level of significance)

### Nervousness and hopelessness

Individuals on the RHU also report the highest levels of nervousness and hopelessness. Measures also show elevated levels of nervousness in TC units, potentially reflecting the added demands of participation in treatment. About 1 in 10 respondents overall reported having experienced no happiness over the preceding week, reflecting an environment perceived as bleak by many incarcerated people.

### Victimization

There are no statistically significant differences between the units in whether individuals had been abused verbally or physically by fellow incarcerated individuals. In contrast, the RHU stands out with elevated reported levels of both verbal and physical abuse by staff. Residents on the honor block reported notably lower levels of verbal abuse by staff.

### Respect and other environmental factors

As shown in panel B of Table 1, results from this administration of the PCQ provide insight into how incarcerated people, as a group, experience various aspects of the prison environment. Levels of respect between incarcerated people, as well as perceptions of how staff acted, including how motivating, respectful, or engaging they were, were consistently higher on the Honor Block.

### Connection with their communities on the outside

Results also indicate how incarcerated people felt about their non-carceral communities and their ability to communicate with them. Respondents generally agreed with the statement that they had a good support system on the outside. Between-unit differences emerged, however, when considering how often respondents were able to connect with those individuals. Perhaps unsurprising given the restrictions imposed upon them, individuals in the RHU reported less access to visits, calls, and messages. Taken together, these factors highlight the increased degree of isolation that can be found in various living units within the same facility.

### Safety and fear

Individuals reported varying degrees of safety and feelings of fear, with slightly higher reported levels of safety on the Honor Block. Given the other results, the relatively low levels of reported fear are noteworthy. The items on the PCQ regarding fear tap into very distinct feelings of vulnerability; the low scores may reflect the wording of specific items on the PCQ. Consistent with results on staff-prisoner relationships discussed above, people in the RHU reported higher rates of fear of staff; rates of discrimination are lowest on the Honor Block.

## Discussion

The impact of incarceration on people and communities, especially those with a history of marginalization, is an issue of health as much as one of justice [10]. The carceral system sits at the nexus of a complex set of societal and justice-related factors. Despite recognition that what happens to people while incarcerated—and how they experience it—is relevant for criminological outcomes such as recidivism and reentry, our understanding of which aspects of that experience may be relevant for health policy largely remains opaque.

The results of the current analysis provide new insight into the lived experience of American incarceration, particularly for marginalized groups who are often overrepresented. For example, studies have found that black individuals experience an increased likelihood of being sent to solitary confinement as a sanction [35], a placement generally associated with negative impacts on health and higher rates of self-harm [36]. The self-reported, relatively high degree of hopelessness, isolation, and fear described by individuals in the RHU here provides contextualization for these findings. They also highlight opportunities to develop place-specific interventions to limit those effects and assess the positive and negative impacts of these changes from the perspective of those most directly impacted [37]. For example, evaluations of TCs similar to those in this study have shown effects that vary by outcome measure employed [38], and are subject to difficult-to-measure influences such as the social networks on the unit [39]. Systematic assessments of prison climate, from the perspective of residential program participants and not coarse administrative records, may prove useful in better understanding how unit-specific context may mediate or moderate programmatic efficacy.

At the facility level, assessments of climate can help us to understand the stratification of the experience of incarceration. Because some measures are more consistent across units (including access to healthcare and other services), they may reflect a divergence between the aspects of incarceration tied to the housing unit. They contrast with those managed at the facility level—a constant for all incarcerated people. This supports framing the prison environment as a multi-level construct and has implications for how health and justice policies are implemented, experienced, and evaluated.

The between-unit differences observed may reflect, at least in part, the interaction between the population composition of the units and the unit-specific environments in which data we collected data. The Honor Block, for example, disproportionately, though not exclusively, housed older individuals serving longer sentences, whereas Therapeutic Communities housed a population struggling with substance use. Reports by people on the Honor Block that they receive better support from staff than those in Therapeutic Communities may reflect the relative ease of maintaining good relationships with a more stable population, as much as reflecting the perceived quality of staff assigned to these units. Differences may also reflect time-varying factors that precede an individual’s unit assignment; the onset or deepening of mental health problems often precede a move to the RHU. These factors combine with the effects of the unit-level characteristics of the built environment and the unique climate and relationships within each unit.

Collection of longitudinal data and causal analyses following changes in unit assignment would facilitate a clearer view of the unique impact of unit climate, better controlling for these factors. Disentangling population-level characteristics from factors within the control of the prison administration is; this is an important avenue for future research. These results provide an assessment of a wide range of carceral experiences, even though we collected these data in a single correctional institution. Self-reported mental health indicators and victimization by staff also varied meaningfully across units, whereas self-reported physical health and indicators on access to health care varied little across units. Linked to an understanding of carceral experiences, self-reported health data gathered in prison climate surveys can inform holistic, targeted, and context-specific interventions for carceral populations. For example, data on sleep quality can inform the deployment of targeted environmental remediation, including sound dampening panels and “quiet time” policies, while responses regarding meals and commissary can assist in developing a culinary program that is both healthier and better received than many standard prison offerings.

Survey results derived from the PCQ and other instruments can also be used to address empirical gaps. First, they can corroborate administrative data—the foundation of most prison-focused inquiries. Researchers will be able to compare administrative records on violence, conflict, and assaults to survey data, for instance, to provide insight into potential underreporting of victimization [23]. Second, they can supplement administrative data about those elements of climate most closely linked with health. Prison records shed light on security and service utilization rather than the well-being and perspectives of incarcerated people. Where the use of force by staff generates an incident report, no administrative data cover how often people are yelled at or the prevalence of stressful, but non-violent, conflicts. Underreporting by victims in prison may be an issue in sensitive matters [40], potentially limiting the utility of administrative data alone in characterizing key experiences of incarcerated people. Similarly, while administrative data will show the levels of service usage (e.g., visits to the dentist), they do not always capture satisfaction with the treatment received. As noted previously, this type of extant data, even when captured, is often difficult to access due to regulations. For example, in the US, the Health Insurance Portability and Accountability Act (HIPAA) broadly restricts access to many health records. The protected nature of the institutions and populations involves other pragmatic restrictions. Third, when the two types of data are linkable, they can be complementary and used for internal verification, evaluations of the intended and unintended consequences of punishment and treatment, and for benchmarking facilities on more administrative functions such as supporting accountability and policy reform [34]. Beyond contextualizing the general prison environment, climate surveys can provide a foundation for program development, implementation, and evaluation. When linked to an intervention or a specialized housing unit, these data can provide otherwise lacking insight into how treatment is being perceived by participants and provide important contextualization for considering effectiveness, especially when self-motivation is paramount [41]. In this facility, the therapeutic community model used on some units inextricably links housing to treatment access and quality. Climate measures may help to explain some of the complex findings in evaluations of these programs [42].

## Conclusions

Measures of prison climate present an opportunity to peer into the “black box” of imprisonment and better understand differing ways people experience incarceration within complex carceral systems [2, 15, 18]. This understanding, fusing concepts of discrimination, access, and equity, in addition to health, provides an opportunity to consider aspects of the carceral experience in a manner that reflects our more robust understanding of the impact of those same policies and practices on health and safety in the community.
